# Artificial intelligence and algorithmic bias: implications for health systems

**DOI:** 10.7189/jogh.09.020318

**Published:** 2019-12

**Authors:** Trishan Panch, Heather Mattie, Rifat Atun

**Affiliations:** 1 Department of Health Policy and Management, Harvard T.H. Chan School of Public Health, Harvard University, Boston, Massachusetts, USA; 2 Wellframe, Boston, Massachusetts, USA; 3 Department of Biostatistics and Executive Director, Health Data Science Masters Program, Harvard T.H. Chan School of Public Health, Harvard University, Boston, Massachusetts, USA; 4 Department of Global Health and Population, Harvard T.H. Chan School of Public Health, Harvard University, Boston, Massachusetts, USA

Artificial intelligence (AI) is a family of techniques where algorithms uncover or learn associations of predictive power from data. An algorithm is a step-by-step procedure for solving a problem. The most tangible form of AI is machine learning, which includes a family of techniques called deep learning that rely on multiple layers of representation of data and are thus able to represent complex relationships between inputs and outputs. However, learned representations are difficult for humans to interpret [1].

AI is a potentially transformative tool for improving inference from data for care and population health [1]. However, while AI has demonstrated substantial potential in clinical applications [2], few large-scale deployments exist, and there are concerns [1]. First, AI is a misleading term. In practice it is more *A* than *I*. It is a defined process applied to ‘narrow inference tasks’ where large volumes of data are present and processing power is available to find associations. It is not, yet, a “general purpose” replacement for human intelligence or ingenuity. Second, whilst there are encouraging research findings in the use of AI in health care, little of this work has been applied in practice, rigorously evaluated or exposed to peer-reviewed publications, while widely publicised positive findings have been challenged [3]. Third, where AI has been used in the broader economy, concerns have emerged regarding its negative consequences in relation to ‘bias’: where AI could amplify inequities in society. For example, in the United States more African Americans have been denied loans or granted longer prison sentences compared to their Caucasian counterparts [4]. For many, the concern is not only that “algorithms are for the most part reflecting back the bias in our world” [5], but that they are doing so at potentially massive scale and without due oversight. Collectively, these shortcomings produce ‘algorithmic bias’, which at present, is not defined in the context of health systems.

We define, for the first time, algorithmic bias in the context of AI and health systems as: “the instances when the application of an algorithm compounds existing inequities in socioeconomic status, race, ethnic background, religion, gender, disability or sexual orientation to amplify them and adversely impact inequities in health systems.”

## AI AND ALGORITHMIC BIAS IN HEALTH SYSTEMS: CHALLENGES

There are three challenges health systems will face in addressing algorithmic bias. First, lack of a clear definitions and standard of “fairness”, second, insufficient contextual specificity, and third, the “black-box” nature of algorithms.

### Lack of a clear standard of fairness

A consumer study of an image search on a popular search engine revealed that 11% of results for the term “CEO” were female [6]. At the time, 20% of CEO’s in the US were women [7]. Were the algorithms in question biased or were they reflecting the data available? Inherent is the challenge – systematic inequities are embedded in societies and health systems and it is difficult to define a standard of fairness in a general way. Algorithms are trained on data from the world as it is which creates the need for additional stewardship, which is complicated as there is no broadly recognized quantitative summary metric for fairness and hence evaluation is ultimately qualitative, and subject to implicit biases of the evaluators.

### Lack of contextual specificity

Health systems vary in design, objectives and the diversity of groups of people they serve from different cultures and environments, with different socio-economic profiles, lifestyles, preferences and genetic endowments. A “generally applicable” AI model should be developed on data reflecting this rich diversity. However, in reality the data are not uniformly available for all socioeconomic groups. Hence, an imbalance in socio-economic or other ‘class’ categories – ie, a certain group or groups are not sampled as much as others or at all – produces insufficient data to accurately make predictions for underrepresented groups.

### The black-box nature of deep learning

Deep learning involves the transformation of data from the real world, such as the pixels of an x-ray, into multiple layers of numbers that are combined to create an output of a diagnostic category. In practice there are up to 100 layers and the relative influence of different elements in each layer is established in the process of learning. This byzantine process yields powerful results, but exactly how it does so is difficult to establish. Data scientists, clinicians and patients want, need and have the right to know how an algorithm produced a particular outcome or prediction.

## ACTIONS TO COUNTER THE RISK OF ALGORITHMIC BIAS IN HEALTH SYSTEMS

To deliver positive impact any AI-driven clinical or policy intervention needs trust of individuals who contribute their data and whose lives are affected, and from clinicians and policy makers who may use AI to inform decisions. Building such trust by addressing algorithmic bias requires several interventions.

### Establish context in which algorithms will be developed and deployed

Ultimately, if bias is present in the world it will be present in the data and will be learned in some form by machine learning algorithms. Simply hiding or protecting certain variables will not be sufficient for reducing algorithmic bias, for their influence will persist in the data [8].

Algorithmic bias is not just a technical issue. Framing it as such will beget an engineering solution. The data inequities that generate algorithmic bias are the same as those that determine who falls ill, whom accesses care, who are represented in data sets used in health systems, how they are treated and who survives. Hence, teams developing algorithms should be explicitly aware of the specificities of the health system context for which they are developing algorithms, by considering differential needs of different groups – best achieved through multi-disciplinary data science teams and by appropriate regulation and evaluation of algorithms and the data science process itself.

### Establish processes to counter the risks of bias in algorithm development

Without intervention, the default technocratic approach in algorithm development will be optimization for algorithmic performance. Often, there will likely be a trade-off between the speed of algorithm deployment and algorithmic bias. A reasonable control mechanism to counter this trade-off is to create “human-in-the-loop” systems, where algorithmic outputs are passed to a human decision maker with necessary caveats and the human is the ultimate decision maker [9].

Training of algorithm developers and data scientists to recognise and consider bias will help reduce it in algorithm development, and where algorithms are published in academic journals the peer review process can be helpful [8]. But given the speed of development, it is unlikely that in future the majority of algorithms will be validated through publications.

### Balanced development of the discipline of health data science

The process of developing AI algorithms is both an art and a science. Data science teams rely on engineers and statisticians, professions where there are known issues regarding gender and racial diversity. There is a critical need for data scientists in health systems and the development of graduate training in health data science is timely, as is the development of the “Data Scientist Oath” that enshrines a specific commitment to addressing algorithmic bias [10].

In AI, the choice of data, algorithm, performance measures and analysis of algorithmic outputs to optimize performance and minimize bias requires considerable judgment. Where possible, data science teams should be as diverse as the populations that the AI algorithms they develop will affect. Specifically, the interests, skills, and life experiences of underrepresented minority populations are relevant in identifying potential sources of bias, as diverse teams will be more intimately familiar with the challenges faced by those who are underrepresented in data sets or unfairly targeted by algorithms. However, diversity alone will not eliminate implicit bias in data science teams. Awareness and sensitivity to implicit biases and their influence on decision making is as important for data science teams as it is for clinicians and policy-makers.

As with diversity of representation, diversity of discipline and patient representation is also important. In particular, clinicians in data science teams offer particular value through their domain expertise to focus data science efforts on problems that are ‘relevant’, and not just ‘technically interesting.’ Clinicians can offer insights into the clinical process that created the data and use their expertise in the art of ‘feature engineering’, which involves selecting predictors (or features) for a model and transforming existing data into a format an algorithm is better suited to handle.

### Transparency and explainability in algorithm development

Whilst it is difficult to explain the inner workings of a deep learning algorithm to a non-expert, the disclosure of algorithmic inputs, the algorithm and its parameters, algorithmic outputs, and counterfactuals used will offer the necessary transparency for their deployment. This transparency would enable clinicians and policy-makers to know which information is being used to make a decision and what the other possible outcomes were, giving them the resources to make informed choices regarding the utility of algorithmic recommendations.

**Figure:**
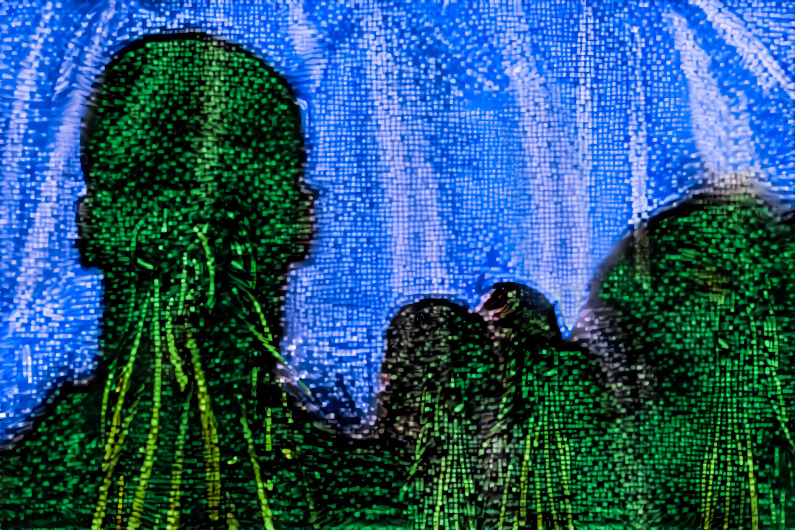
Photo: Artificial intelligence and algorithmic bias: Beware of averages while ensuring diversity and inclusion (Credit: iStock).

Several methods have been proposed to increase ‘explainability’ and identify bias, with researchers attempting to reverse-engineer the drivers of bias. This involves using the inputs (if known) and outputs of an algorithm to discover how the algorithm made its decisions. This process is difficult, time consuming, and non-intuitive to non-experts of machine learning methods [11]. What might be a more practical approach is using clinical expertise to propose relevant counterfactuals for the context in which the algorithm is being developed [12]. Here, a counterfactual is created when a change in one of the inputs corresponds to a change in the output, or decision, of the algorithm. Where income, race, etc. are modified and a different outcome is produced, either there is an instance of bias due to misclassification, or broader social determinants need addressing to reduce bias.

### The role of the public sector in AI and in countering the risk of algorithmic bias in health systems

The public sector has an important role in countering the risk of algorithmic bias. First, by establishing standards of fairness [13]. Second, regulating algorithms that will be deployed in health systems [14]. Third, by introducing mechanisms to address emerging issues, where algorithms uncover known or unknown biases in society, reflecting structural inequities, or illuminate biases in clinical decision making reflecting implicit biases of clinicians. Fourthly, by encouraging conducive partnerships between public and private sectors to ensure development of AI to benefit all. Some technology companies have invested large sums in not just AI algorithms but in the computing infrastructure necessary for their creation or deployment – assets that could be harnessed by public sector [15]. However, large data sets needed for AI require public infrastructure to create them especially with necessary representation to ensure equity. These data sets could be made available to those developing algorithms with protection of patient privacy and fair attribution of any financial rewards to benefit both public sector organisations, which care for patients and populations and generate data, and private sector organisations that have the expertise to use of the data to create AI algorithms.

## CONCLUSIONS

We define algorithmic bias in health systems, a risk inherent in AI, explore its implications for health systems and identify ways to mitigate it. If bias exists in society it will both manifest in health systems and be represented in algorithms. The presence of algorithmic bias should however not be the end of the discussion on the application of AI in health systems but the beginning of a new one on how algorithms can be developed in a way that minimizes bias and also how health systems eliminate the deeply entrenched inequities algorithmic bias may further reveal.
